# Controllable interlayer space effects of layered potassium triniobate nanoflakes on enhanced pH dependent adsorption-photocatalysis behaviors

**DOI:** 10.1038/s41598-018-24898-8

**Published:** 2018-04-26

**Authors:** Qinglin Deng, Mengjiao Li, Junyong Wang, Kai Jiang, Zhigao Hu, Junhao Chu

**Affiliations:** 1 Key Laboratory of Polar Materials and Devices (MOE) and Technical Center for Multifunctional Magneto-Optical Spectroscopy (Shanghai), Department of Electronic Engineering, East China Normal University, Shanghai, 200241 China; 2 Collaborative Innovation Center of Extreme Optics, Shanxi University, Taiyuan, Shanxi 030006 China

## Abstract

Despite the extensive study of two-dimensional layered KNb_3_O_8_ (KN), there still remains some vital problems need to be clarified for future applications in environmental purification. Here we demonstrated the successful preparation of interlayer-controlled KN nanoflakes using alkaline hydrothermal conditions by adjusting the amount of thiourea in the reaction. This process resulted in KN nanoflakes with a larger specific surface area than previously reported. Moreover, the initial pH of dye solution and discrepant preferential orientation of interlayer peak have been proved to significantly influence the adsorption and photocatalysis performances of KN. In addition, relevant photocatalysis mechanisms have been expounded, by combined the first-principles calculation. The present work could be helpful in revealing the intrinsic adsorption-photocatalysis features of KN and other similar niobates.

## Introduction

With the growing demands for solving environmental problems, low dimensional semiconductor materials with multifunctional applications have raised considerable research interest^1,2^. Two-dimensional (2D) layered transition metal oxides should be unnegligible due to their charming features, such as the excellent thermal stability under an oxygen atmosphere^3,4^. As a typical case, layered KNb_3_O_8_ (KN) possesses unique interlayer regions, which makes it of great interest for applications in environmental purification, non-linear optics, energy conversion, etc^5–9^. KN has an orthorhombic crystal structure with an *Amam* space group. It also has a 2D layered structure consisting of linked NbO_6_ octahedral units. K^+^ ions exist between the negatively charged layers to maintain the charge balance^10–13^.

As a typical layered semiconductor, although the photochemical and ion exchange properties of KN have been extensively studied^7,12,14,15^, there still remain some pivotal problems need to be settled for future applications. For the preparation process, KN can be synthesized by various methods, such as solid-state reaction, molten salt synthesis and hydrothermal growth^16–19^. As compared with the conventional solid-state reaction, the hydrothermal method has been less frequently reported. As we know, hydrothermal method is a well-accepted method due to its obvious advantages, such as low cost, controllable morphology and mild reaction conditions^20^. Generally, using hydrothermal method can obtain a much larger specific surface area than using solid-state reaction method. Note that the specific surface area and pore-size distribution of KN would significantly affect its photocatalytic abilities. At this stage, although there are some reports on using hydrothermal method to prepare KN, their specific surface areas are urgently desired to improve. Moreover, most of these hydrothermal experiments were conducted under acidic conditions^13,21–23^, which promoted an ion exchange reaction between K^+^ and H^+^. It was noteworthy that, our previous study has demonstrated that layered K_4_Nb_6_O_17_ can be successfully synthesized by one step hydrothermal reaction using Nb_2_O_5_ and KOH as the starting materials^20^. Lamentedly, the production rate is unsatisfactory, accompanied by numbers of K and Nb ions fail to react. Thus, it is vital to propose an approach for making full use of the resulting solution to prepare KN under alkaline hydrothermal conditions.

Decomposition of pollutants is a popular application for photocatalytic technologies and a challenging mission^24–26^. TiO_2_-based catalyst was frequently adopted to photodegrade pollutant^27–29^. But the limited efficiency restricts its development. It is urgent to explore other superior materials for dye degradation. Since KN is known to be an effective catalyst, various strategies have been applied to enhance the catalytic performances of KN-based materials^30–32^. Good dye photodegradation properties of KN can be found in some reports^5,22^. However, the influences of pH on dyes adsorption and photodegradation have seldom been investigated. It was believed that controlling the dye environment can be effective in tuning photocatalytic activities^12,26,33^. Furthermore, in order to guide the photocatalytic activities of KN-based and other similar layered semiconductor materials, their relevant photocatalytic kinetic mechanisms should be further clarified.

Basing on the above concepts, herein we devoted much efforts to make full use of the reactive raw materials to prepare KN under alkaline hydrothermal conditions. Remarkably, the quantity of thiourea in the reaction played a key role in the preparation of interlayer-controlled KN nanoflakes. The effects of initial pH of dye solution and discrepant preferential orientation of interlayer peak on adsorption and photodegradation have been systematacially investigated, as well as the relevant kinetics mechanisms. It is believed that the present work shows novel insights into KN and could be helpful in developing potential multifunctional KN-based applications.

## Results and Discussion

### XRD, Raman, BET, TGA and morphology analysis

**Figure 1:**
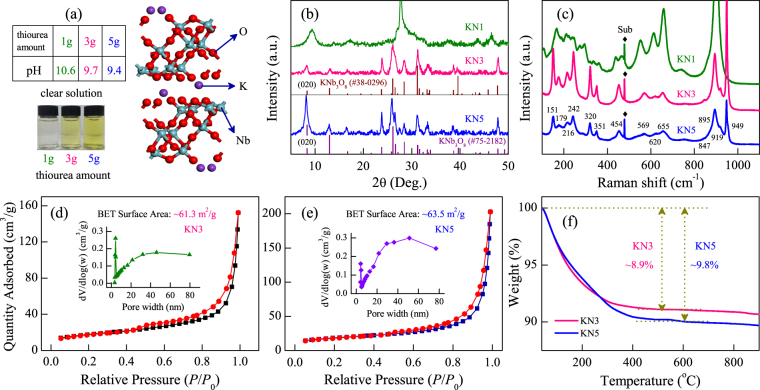
(**a**) Simulated structure of KN. (**b**) XRD patterns and (**c**) Raman spectra of KN1, KN3, and KN5. Nitrogen adsorption-desorption isotherm and pore-size distribution (inset) for (**d**) KN3 and (**e**) KN5. (**f**) Thermogravimetric analyses of KN3 and KN5.

The supercell of KN orthorhombic structure was shown in Fig. 1a. As we can see, the unique layered structure of KN is composed of (Nb_3_O_8_)^−^ layers and K^+^ ions occupy the interlayer regions. In the work, it was found that the different amount of thiourea plays a key role in the preparation of KN. The real color contrast of the resulting solution after the hydrothermal reaction with different amount of thiourea have been presented in the inset of Fig. 1a. As we can see, the solution is nearly colourless by adding 1 g of thiourea. It turns into yellow after adding 3 g of thiourea, then the color deepen when increasing the amount to 5 g. It is noteworthy that all of the solutions are alkaline, and the pH values are summarized in the table of Fig. 1a.

Figure 1b shows the X-ray diffraction (XRD) patterns of as-prepared samples. As we can see, KN1 was the orthorhombic phase K_4_Nb_6_O_17_. The diffraction peaks of KN3 and KN5 can be assigned to the orthorhombic phase of KNb_3_O_8_ (JPCDS No. 38-0296 or 75-2182). Interestingly, the intensity of the (020) diffraction peak for KN5 is higher than that of KN3, indicating that KN5 was preferred orientation along the (020) peak to grow. Note that KN possesses interlayer spaces alternately between (Nb_3_O_8_)^−^ layers, which can adsorb moisture in air. The (020) diffraction peaks are characteristic of the layered structures of KN. Thus, we demonstrated that the interlayer spaces of KN can be controlled by adding different amount of thiourea. The remarkable variation can affect the photochemical properties (seen in the subsection of Adsorption and Photodegradation Activities for Dyes).

As typical examples, Raman scattering spectra of KN1, KN3 and KN5 were shown in Fig. 1c. It can be seen that KN1 shows sharp and strong Raman bands around 901 cm^−1^. A detailed discussion of the Raman spectra for KN1 can be found in our previous study^3^. Theoretically, KN has an orthorhombic structure with an *Amam* space group. The NbO_6_ octahedra are the main contributors to the stretching and bending vibrations modes. As depicted by Fig. 1c, the Raman bands of KN3 and KN5 were basically the same except for the intensity. Detailedly, the Raman bands at 949, 919, 895, 847 cm^−1^ and 655, 620, 569 cm^−1^ were assigned to the stretching modes of the shorter and longer Nb-O bonds, respectively. The remaining modes in the 151–454 cm^−1^ range correspond to bending vibrations of the NbO_6_ octahedra.

The specific surface area and pore-size distribution of KN3 and KN5 were investigated using N_2_ adsorption-desorption isotherms at 77 K. As shown in Fig. 1d and e, the BET specific surface areas of KN3 and KN5 are about 61.3 and 63.5 m^2^ g^−1^, respectively. Both of them exhibit a characteristic isotherm of type IV. The adsorbed volume of KN3 and KN5 gradually increases with an evident hysteresis loop (0.5 < *P*/*P*_0_ < 1). The insets of Fig. 1d and e indicate that KN3 and KN5 also have a wide range of pore size distributions. Remarkably, the KN nanoflakes prepared in our work provide a relatively larger BET surface area than previous typical reports^12,13,22^. Consequently, it was expected that KN3 and KN5 would exhibit superior photocatalytic activities due to their high BET surface area.

The stability of KN3 and KN5 were confirmed by TGA, as shown in Fig. 1f. Both of two samples exhibit one distinctive weight loss stage due to the evaporation of adsorbed water. The total mass losses of KN5 and KN3 are about 9.8% and 8.9%, respectively. As compared with KN3, KN5 exhibits a higher weight loss. It can be attributed to the larger BET surface area of KN5, which allows more molecule to adsorb on its surface.

**Figure 2:**
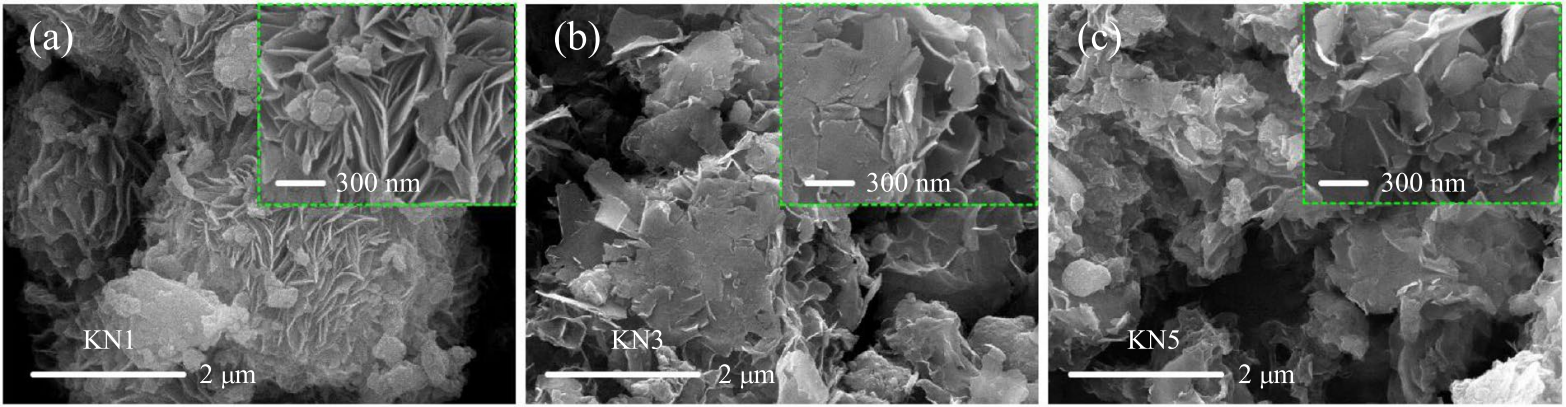
SEM images of (**a**) KN1, (**b**) KN3, (**c**) KN5 under different magnifications.

The morphologies of the as-synthesized samples were characterized by scanning electron microscopy (SEM). As shown in Fig. 2a, KN1 exhibits aggregated lamina morphology from different magnifications. Both of KN3 and KN5 have the similar morphologies. As we can see, they are composed of thin nanoflakes, which pack densely and show a porous structure, as shown in Fig. 2b and c.

### Adsorption and photodegradation activities for dyes

**Figure 3:**
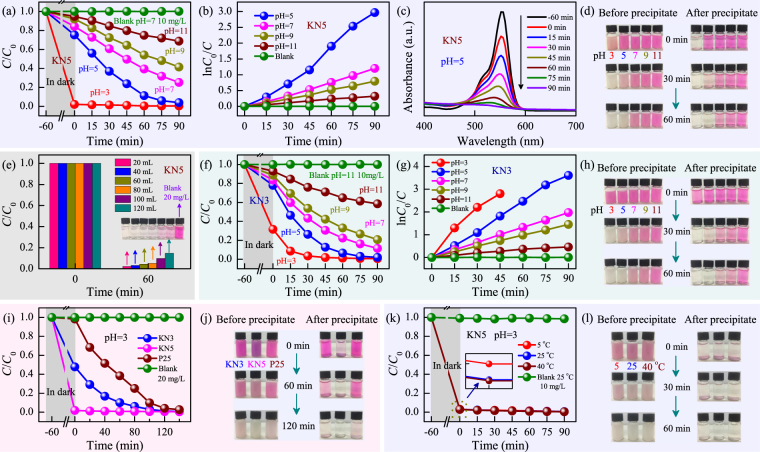
(**a**) The adsorption (in dark) and photodegradation (under light irradiation) effects (*C*/*C*_0_) for KN5 in the RhB aqueous solution (10 mg L^−1^) with different pH values. (**b**) The plots of ln(*C*_0_/*C*) *versus* irradiation time *t* for KN5. (**c**) The corresponding adsorption spectra for KN5 when pH = 5. (**d**) The real color contrast of the relevant RhB dye solution for KN5. (**e**) The adsorption capacity of KN5 (20 mg) for the RhB solution (20 mg L^−1^, 20–120 mL) when pH = 3. The insets show the color contrast of the relevant RhB dye solution. (**f**) The adsorption and photodegradation effects for KN3 in the RhB aqueous solution (10 mg L^−1^) with different pH values. (**g**) The plots of ln(*C*_0_/*C*) *versus* irradiation time *t* for KN3. (**h**) The real color contrast of the relevant RhB dye solution for KN3. (**i**) The adsorption and photodegradation effects for KN3, KN5 and P25 in the RhB aqueous solution (20 mg L^−1^) when pH = 3, with (**j**) the real color contrast of the RhB dye solution. (**k**) The adsorption and photodegradation effects for KN5 at different temperatures in the RhB aqueous solution (10 mg L^−1^) when pH = 3, with (**l**) the real color contrast of the RhB dye solution.

Dyes adsorption and degradation is an interesting topic for efficient treatment of dye effluents^34–36^. In order to better evaluate the adsorption and photodegradation performances of KN, in the work, the well-known rhodamine B (RhB) dye was adopted as the organic contamination. Note that all of the mixed dye suspensions must be stirred in the dark for 1 h to establish an adsorption-desorption equilibrium. It serves two purposes, one is to investigate the adsorption effects, the other is to exclude the interference from absorption during light irradiation. The adsorption and photodegradation efficiency is defined as (*C*_0_−*C*)/*C*_0_, where *C* and *C*_0_ denote the real-time and initial concentration of dye solution, respectively. It is believed that pH is an important factor that influences the adsorption and photocatalytic performances of a catalyst. As shown in Fig. 3a, the adsorption efficiency of KN5 increased with decreasing the pH values of dye suspensions. In detail, when pH was 5, 7, 9 and 11, the adsorption efficiency was about 25%, 16%, 8% and 5%, respectively. Unexpectedly, it achieved a ultrahigh value of approximately 98% when pH decreases to 3. Due to the influences from pH, the photodegradation efficiency of KN5 dramatically increased with decreasing the pH values. As we know, the degradation rate of dyes can be obtained by the Langmuir-Hinshelwood (L–H) equation. It was expressed as ln(*C*_0_/*C*) = *kt* + A, where *k* and *t* denote the degradation rate and reaction time, respectively. As evident in Fig. 3b, the photodegradation process of KN5 were well fitted into a pseudo-first order reaction. The *k* value can be extracted according to the slope of curves. Figure 3c shows the adsorption spectra of RhB solution in the presence of KN5 during different time. As we can see, the main absorbance of the RhB solution was located at ~553 nm. The intensity has a significant reduction, and the spectra show a slight blue shift. In view of the superior adsorption feature of KN5, especially under low pH condition, the real color contrast before and after precipitate for the RhB dye suspensions have been presented in Fig. 3d. Although KN5 has an excellent adsorption efficiency, as demonstrated by the nearly colorless dye solution when pH was 3, the RhB dye was not degraded by KN5. Fortunately, the catalyst can return to its initial color as the light irradiation time was extended, indicating the successful removal of RhB by photodegradation. The color contrast under other pH conditions were in accord with the results in Fig. 3a. KN5 shows admirable adsorption performances under low pH conditions for RhB solution. It is vital to research the maximum adsorption ability for potential applications. Figure 3e shows the adsorption effects of KN5 (20 mg), for the RhB dye solution (20 mg L^−1^, 20–120 mL) when the pH value was 3. As we can see, the adsorption efficiency slightly decreased with increasing the volume of dye solution. Concretely, when volume was 20, 40, 60, 80, 100 and 120 mL, the adsorption efficiency was about 98%, 97%, 96%, 95%, 90% and 85%, respectively.

Moreover, the adsorption and photodegradation performances of KN3 in RhB dye solution have been also investigated, as shown in Fig. 3f. Due to the preferential orientation of (020) peak between KN5 and KN3, they show the different adsorption and photodegradation behaviors. The adsorption efficiency of KN3 was about 68%, 22%, 17%, 10%, and 7% for the pH value at 3, 5, 7, 9 and 11, respectively. This phenomenon was similar to KN5 with the exception of the performance of KN5 at pH = 3. Figure 3g indicates that a pseudo-first order reaction was also acceptable for KN3. Interestingly, the photodegradation rates of KN3 were slightly higher than the values of KN5 except for pH = 3. The reasons might be attributed to the discrepant crystallization orientation and Raman bands. Figure 3h also presents the real color contrast before and after precipitate for KN3 under different pH conditions, which is similar to the results of KN5. In addition, KN5 shows superior adsorption and photodegradation activities for other organic dye molecules, such as methylene blue (MB) and methyl violet (MV) (not shown), which are similar to the results of RhB dye.

In consideration of the superior adsorption-photocatalysis performances of KN3 and KN5, it is of great significance to qualitatively compare the photocatalytic ability with other catalysts. To accomplish this, commercial catalyst (TiO_2_, Degussa P25) was used as photocatalytic comparison material, as shown in Fig. 3i. Note that the pH values of all test suspensions were adjusted to 3. For the adsorption efficiency, KN5 maintains a high value of about 98% when the concentration of the RhB solution is increased by one fold. However, the adsorption efficiency of KN3 is about 52%, which is lower than that at the concentration of 10 mg L^−1^. This phenomenon indicates that KN3 has the limited adsorption capacity for RhB dye. Combining with the real color contrast (shown in Fig. 3j), it was found that KN3 required more time than KN5 to completely photodegradate RhB. Moreover, P25 shows negligible adsorption activities, and the photodegradation rate is also lower than KN5. In addition, the adsorption and photocatalysis experiments for KN have been carried out at a lower and higher temperature, as shown in Fig. 3k and l. The results indicates that the temperature of dye solution can hardly influence the photocatalytic activity of KN. When the temperature is ~5°C or 40°C, KN5 also shows good photocatalytic performances. It has widened the working conditions of KN. Note that the blank experiments without a catalyst show negligible adsorption and photocatalytic activities, which indicates that the degradation reaction are truly driven by a photocatalytic process. For KN, most of the previous reports have ignored obviously the ability of adsorbing dyes and the influence derived from the pH of dye solution. Remarkably, it can be concluded that KN5 prepared in our work shows excellent photocatalytic activities with the aid of adsorption effects.

### Relevant kinetics mechanisms

**Figure 4:**
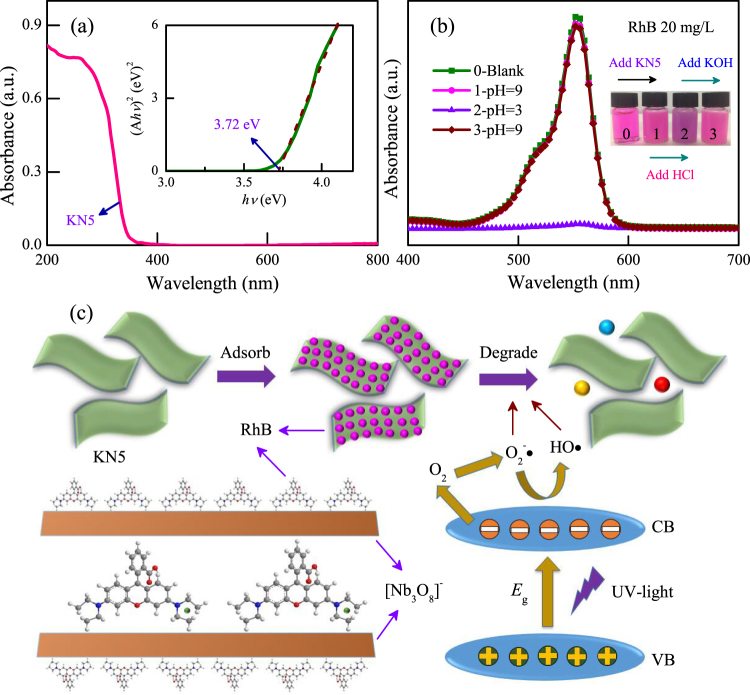
(**a**) UV-VIS DRS of KN5 with the inset curve of (*Ahν*)^2^ *νs*. *hν*. (**b**) The adsorption spectra of RhB solution under changed pH conditions for KN5. The inset shows the color contrast of the mixed dye suspensions. (**c**) Relevant kinetics mechanisms of adsorption and photocatalysis for KN5 in RhB solution.

In order to further guide the photocatalytic performances of KN and other layered oxides, it is essential to making out the relevant kinetics mechanisms. As we know, the photocatalytic activities of semiconductors was deeply affected by the band gap structures. As a typical example, Fig. 4a shows the UV-VIS diffuse reflectance spectra (DRS) of KN5. The spectra clearly shows the characteristic absorption edge. The band gap of KN5 can be obtained from the plots of (*Ahν*)^2^ *νs*. *hν*, which is estimated to be 3.72 eV. Moreover, the adsorption spectra of RhB solution at various pH values were shown in Fig. 4b. As we can see, the color of mixed dye suspensions did not change when adding KN5 into raw RhB solution. However, the color did change when the pH value of dye suspension was adjusted to 3. Fortunately, the dye suspension can return to its initial color when the pH value is returned to a value of 9, and all of the spectra exhibit the characteristic absorption peak of RhB dye. This phenomenon indicates that the RhB dyes were not damaged or decomposed by KN5. In addition, Fig. 4c vividly depicts the mechanisms of adsorption and photocatalysis. The RhB molecules will be ionized under low pH condition. Due to electrostatic attraction and interlayer space of layered KN, RhB dye molecules can be adsorbed onto/into KN until a charge balance is attained. The adsorbed dyes are then quickly photochemically degraded by light irradiation. Furthermore, as KN has a high adsorption capacity for dyes, the RhB dyes would be continuously supplied as a result of the concentration difference between the interface layers of KN and dye solution. Finally, as the reaction continues, most of dyes can be completely degraded. Thus KN5 displays the better photocatalytic activity than KN3 owing to its better adsorption ability. It is believed that the mechanism can be helpful in exploring the photocatalytic activities of other similar layered materials.

### Theoretical study

In order to further explore the electronic structures of KN and explain the relevant experimental findings, the plane-wave-based density-functional theory (DFT) calculation with the generalized gradient approximation (GGA) was carried out for orthorhombic phase KN^10^. The Perdew-Burke-Ernzerhof (PBE) functions are used to address exchange-correlation interactions along with a standard plane-wave basis set with a kinetic-energy cutoff of 340 eV. These calculations are performed by using the 2 × 1 × 4 Monkhorst-Pack *k*-point mesh, and the convergence criterion for the electronic energy is 10^−5^ eV.

**Figure 5:**
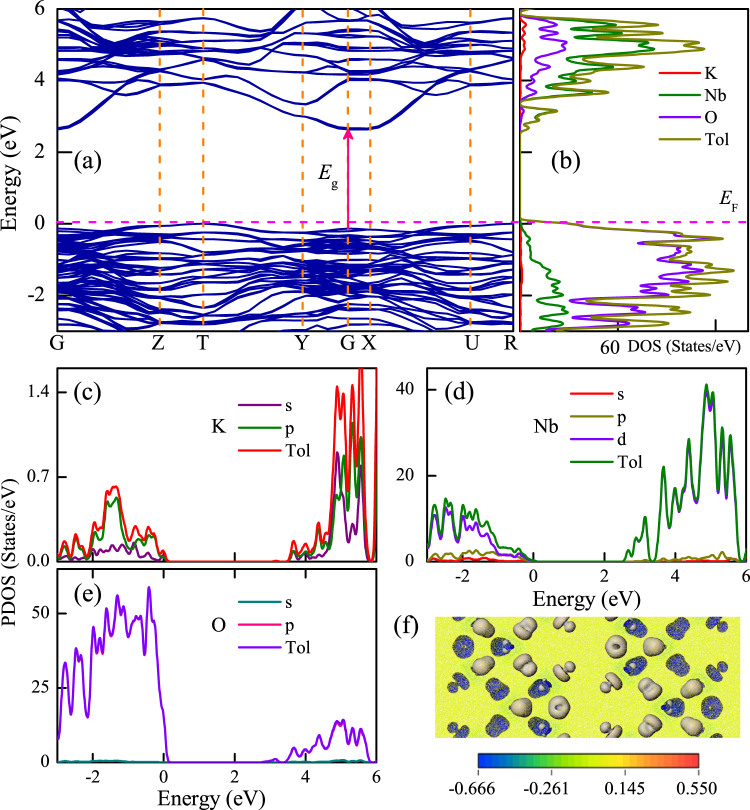
(**a**) Calculated band structure and (**b**) corresponding density of states for KN, where *E*_F_ denotes the Fermi energy level. Note that the solid arrow located at the point of symmetry *G* indicates the direct electron transitions of optical band gap. Partial density of states for (**c**) K, (**d**) Nb and (**e**) O elements, respectively. (**f**) Charge density difference of KN.

The calculated electronic band structure and density of states (DOS) for KN were shown in Fig. 5a and b, respectively. Note that the highest occupied state was chosen as the Fermi level (*E*_F_) and set to zero as the reference. It was found that KN has the direct electron transition feature. The calculated band gap was about 2.63 eV, which is smaller than the experimental results (3.72 eV for KN5). The reason can be ascribed to the well-known problem of calculations based on DFT. Generally, as compared to the experimental band gaps, the calculated values could be underestimated by about 20%–30%. Moreover, Fig. 5c–e show the partial density of states (PDOS) of K, Nb and O elements. As we can see, the d states of Nb, and the p states of O play a key role in the valence bands (VBs) and conduction bands (CBs). However, the s and p states of K have little contribution to the VBs and CBs. On the basis of DOS and PDOS results, obviously, the top of VBs and bottom of CBs are composed of mainly O-2p and Nb-4d states, respectively. For the photocatalysis experiments, the theoretical study indicated that the photo-induced electrons were excited from O-2p states to Nb-4d states. In addition, as shown in Fig. 5f, the charge density difference of KN clearly presents the strong interaction of Nb and O atoms due to the overlap of atomic orbitals.

## Conclusion

In summary, we have successfully synthesized KN nanoflakes under alkaline hydrothermal conditions with a larger specific surface area. The interlayer spaces of KN can be controlled by adding different amount of thiourea. KN with discrepant preferential orientation of (020) peak show different adsorption and photocatalysis performances, which are also strongly affected by initial pH of dye solution. 5 g of thiourea derived KN shows better photocatalytic activities than commercial Degussa P25. Moreover, relevant kinetics mechanisms and theoretical calculation further reveal the intrinsic photocatalytic features and electronic structure of KN. The alkaline hydrothermal conditions, controllable interlayer space effects, and influences of pH and temperature, reveal novel insights into KN. Therefore, it was concluded that our present work for KN could be helpful in exploring the optoelectronic properties and broadening the multifunctional applications of KN and other similar layered niobates.

## Methods

### Preparation of KN nanoflakes

All of the chemical reagents used in the work were purchased from commercial sources and without any further purification. A novel hydrothermal method was adopted to synthesize KN nanoflakes. Typically, 2.5 g of Nb_2_O_5_ powder was added into 80 mL 1.0 mol L^−1^ KOH solution under stirring for 1 h. The resulting mixture was transferred into a polytetrafluoroethylene-lined stainless autoclave and hydrothermally treated at 200 °C for 15 h, followed by natural cooling to room temperature. The resulting clear solution was collected for later use. Then 1, 3, and 5 g of thiourea (abbreviated as KN1, KN3, and KN5, respectively) were added into 30 mL of the above clear solution. After stirring for 5 h, this mixed solution was transferred into a polytetrafluoroethylene-lined stainless autoclave and hydrothermally treated at 200 °C for 15 h. The final products were collected by centrifugation, washed with deionized (DI) water and absolute ethanol for several times, and then dried at 80 °C in vacuum for 24 h.

### Characterization methods

The crystal phase structures of all samples were analyzed by X-ray diffraction (XRD, Cu K *α*, D8 Advance, Bruker). Raman scattering experiments were carried out by a micro-Raman spectrometer (Jobin-Yvon LabRAM HR 800UV). The Brunauer-Emmett-Teller (BET) surface areas of the powder samples were measured by a surface area analyzer (TriStar II 3020), and the pore-size distribution curves were obtained using Barrett-Joiner-Halenda (BJH) model. Thermogravimetric analyses (TGA) of samples were performed in a TGA/DSC 1 STAR^*e*^ System (Mettler-Toledo) from 40 °C to 900 °C with a heating rate of 15 °C min^−1^. The surface morphologies of powder samples were examined by field emission scanning electron microscopy (FEI Quanta 400 FEG). Ultraviolet-visible light diffuse reflectance spectra (UV-VIS DRS) were recorded by a double beam infrared-ultraviolet spectrometer (Perkin-Elmer UV/VIS Lambda 950) equipped with an integrating sphere assembly.

### Adsorption and photodegradation of dyes

The adsorption and photodegradation performances of as-prepared samples were evaluated by observing their abilities to adsorb (in dark) and degrade (under light irradiation) the Rhodamine B (RhB) dye. The UV-light source was a 500 W long arc Hg lamp equipped with a filter which only allows UV-light through. The photocatalytic experiments were carried out by a reactor equipped with a cooling water cycle system, which can simultaneously conduct six parallel reactions. In a typical photocatalytic test, the catalyst (50 mg) was dispersed in a 50 mL aqueous solution of RhB dye (10–20 mg L^−1^) with different pH (3–11), then the mixed suspensions were magnetically stirred in the dark at room temperature for 1 h to establish adsorption-desorption equilibrium. After light irradiation, adequate volume of the suspension were extracted and centrifuged at an interval of 15/20 minutes for analysis. Note that the pH of the mixed solution was adjusted using KOH and HCl aqueous solution with the aid of a pH meter (Mettler-Toledo). The adsorption and photocatalysis efficiency were investigated by measuring the change in intensity of the characteristic absorbance of RhB dye using spectrometer (Perkin-Elmer UV/VIS Lambda 950).
